# Evaluation of Different Test-Day Milk Recording Protocols by Wood’s Model Application for the Estimation of Dairy Goat Milk and Milk Constituent Yield

**DOI:** 10.3390/ani11041058

**Published:** 2021-04-08

**Authors:** Vincenzo Landi, Aristide Maggiolino, Angela Salzano, Salvatore Claps, Pasquale De Palo, Domenico Rufrano, Giuseppina Pedota, Gianluca Neglia

**Affiliations:** 1 Department of Veterinary Medicine, University of Bari A. Moro, 70010 Valenzano, Italy; vincenzo.landi@uniba.it (V.L.); aristide.maggiolino@uniba.it (A.M.); 2 Department of Veterinary Medicine and Animal Production (DMVPA), University of Naples Federico II, 80137 Naples, Italy; angela.salzano@unina.it (A.S.); neglia@unina.it (G.N.); 3 Council for Agricultural Research and Economics, Research Centre for Animal Production and Aquaculture (CREA-ZA), 85051 Bella Muro, Italy; salvatore.claps@crea.gov.it (S.C.); drufrano@tiscali.it (D.R.); 4 Italian Breeder Association, 85100 Potenza, Italy; pinapedota@gmail.com

**Keywords:** goat, Wood’s model, lactation curve

## Abstract

**Simple Summary:**

Considering the seasonal trend of milk production due to application of estrus synchronization treatments in autumn or in spring season, as well as to meet meat production and market requirements for religious holidays, one may find it useful to simplify the milk and milk constituent yield quantification during the entire period of lactation. The present work aimed to verify the application of Wood’s model to a different number of test-day milk recording protocols in order to reduce the frequency of National Breeding Association test-day milking record protocol. Wood’s model fit well on goat milk production, showing high *R*^2^ values. Moreover, the milk yield, fat-corrected milk yield, fat yield, and protein yield estimation applying Wood’s model to four recordings (first, second, fourth, and sixth month records) showed results superimposable to International Committee of Animal Recording (ICAR) application and Wood’s model application on test-day records every 15 days.

**Abstract:**

Goats have important social and economic roles in many countries because of their ability to survive and be productive in marginal areas. The overarching aim of this study was to compare the application of Wood’s model to different test-day milk recording protocols for estimation of total milk, fat, and protein yield in dairy goats. A total of 465 goats were used (Garganica, 78; Girgentana, 81; Jonica, 76; Maltese, 77; Red Mediterranean, 76; Saanen, 77). Milk yield was recorded every 15 days throughout lactation of 210 days, for a total of 14 collection days, during both morning and afternoon milking sessions. Milk samples were collected and analyzed for protein and fat. The fat-corrected milk was standardized at 35g fat/kg of milk. Wood models showed high *R*^2^ values, and thus good fitting, in all the considered breeds. Wood model applied to first, second, fourth, and sixth month recordings (C) and ICAR estimation showed total milk yield very close to Wood’s model applied to all 14 recordings (A) (*p* > 0.38). Differently, Wood’s model applied to the first, second, third, and fourth month recording (B) estimation showed great differences (*p* < 0.01). This could be applied for farms that had the necessity to synchronize flock groups for kidding in order to produce kid meat. In farms that apply the estrus induction and/or synchronization for kidding, it would be possible to perform only four test-day milk recordings and to apply the Wood’s model on them in order to obtain the estimation of total milk, fat, and protein yield during lactation for animals inscribed, or to be inscribed, to the genealogical book.

## 1. Introduction

Goats have an important socioeconomic role worldwide in rural communities as they are productive in areas where other agricultural activities are not viable. In fact, they provide a livelihood for rural people, particularly in marginal areas. It has been shown repeatedly that goats produce milk efficiently where feed resources are inadequate to sustain dairy cattle [1], but they are also really important for kids’ production in some particular period. In fact, in the Mediterranean Basin, during some religious holidays (i.e., Islamic, Hebraic, Catholic), it is usual to eat kid or lamb meat, and their market request increases, inducing a lot of farmers to synchronize their flocks for kidding. Goat husbandry was one of the earliest agricultural activities in the entire Mediterranean area, and today goat production contributes both milk and meat from local and introduced breeds.

Actually, farmers have often replaced heritage breeds with worldwide spread counterparts to increase their profitability [2]. However, some researchers showed that local breeds are less productive (lower milk yield) but have better milk characteristics—this favors its use for coagulation and cheese production [3]. Considering the seasonal reproductive behavior of the species, farms are often organized in birth groups, according to the market kids request. This is possible by applying estrus synchronization treatments in autumn or in spring season in order to meet meat production and market requirements for religious holidays [4]. Thus, a seasonal trend of milk production is also recorded, increasing the need of satisfying animal requirements, particularly in some periods of the year.

In Spain, thanks to the use of modern breeding plans, various local goat breeds such as the Murciano-Granadina, the Florida, and the Malagueña have been exploited, demonstrating the importance of production potential and economic of indigenous genetic resources [5]. This is particularly important in Italy, where local breeds are often utilized for designation of origin animal derived food. Although some of these breeds of goat have relatively small numbers in Italy (Jonica, 800; Girgentana, 1700; Red Mediterranean, 2100; Maltese, about 3500) [6], some of them have demonstrated to be an important reservoir of genetic variability also in term of environmental adaptability [7].

Milk production can be graphically represented by the lactation curve, and mathematical modelling and fitting of the lactation curve can be a useful way to analyze dairy goat performance [8,9,10,11,12,13]. Mathematical models used in lactation curve estimation are generally classified as three-parameter models (e.g., Wood’s model) or five-parameter models (polynomial equations). Wood [14] proposed an incomplete gamma function to describe the lactation curve in dairy cows and obtained improved fitting of the ascending and descending phases compared with previous models [15]. Due to the ability of Wood’s model to fit relatively well to a broad range of data [16], together with its relative simplicity and reduced number of parameters, it is the most common model used to estimate lactation curves in different dairy species including cattle [17], sheep [18], goats [1,19,20], mares [21], and donkeys [22]. However, the large variability recorded in milk yield and characteristics among goat breeds [1,19,20] prevent a proper evaluation of animal requirements, impairing animal welfare and production.

Actually, the International Committee of Animal Recording (ICAR) [23] defines the guidelines to measure milk yield in dairy goats—in the case of double daily milking, the average recording interval (days) between two successively test-days for a flock is 30 days, with a range from 28 to 34 days. In some periods of the year, the test-day frequency may be modified to better understand the lactation curve of the animals. Lactation curves are of great help for physiologists, nutritionists, and geneticists for studying and testing hypothesis on the behavior of the mammary gland machinery and, by the point of view of genetics evaluation, the possibility to estimate the performance also in young animals allow to get phenotypic information’s faster at the first test days or by other hand the use of more sophisticated statistical model like as random regression equations [24]. The hypothesis is that mathematical model application can be useful to estimate the correct milk yield on the basis of a lower test-day number for each dairy goat. In light of this, the present work aimed to verify the application of Wood’s model to a different number of test-day milk recording protocols in order to modify the frequency of National Breeding Associations test-day milking records protocol, obtaining at the same time good milk and its constituent yield estimation.

## 2. Materials and Methods

### 2.1. Animals and Management

A total of 465 goats belonging to 6 breeds were utilized in the study: Garganica (n = 78); Girgentana (n = 81); Jonica (n = 76); Maltese (n = 77); Red Mediterranean (n = 76); Saanen (n = 77). The animals were reared in the Council for Agricultural Research and Economics, located in the municipality of Bella (province of Potenza) in the south of Italy, and being part of a genetic conservation/multiplication program and included a genetics pool of all the breeds. The farm is at latitude 407,006,303 and longitude 155,472,126, with 15 °C, wind W at 8 km/h, 63% humidity as mean climatological data in a period between March and October.

After kidding, the goats were maintained with their kids for 3 days in order to guarantee colostrum intake. Only animals with 1 kid were included in the study and further processed. The study was carried out over a 210-day period from late winter to early autumn at the experimental farm. The animals were maintained in paddocks under a free stall system and the same management conditions. In late winter, the goats were maintained indoors in breed groups in free stalls and fed a commercial concentrate and had ad libitum access to mixed-species dry hay (Table 1).

From early spring to late summer, the goats were maintained on the pasture for 8 h/day and returned indoors at night where they had access to commercial concentrate and dry hay. The amount of concentrate was calculated for each breed on the basis of the average metabolic weight and mean milk production of each breed according to the National Research Council (NRC) [25]. This was adjusted every 15 days throughout the study after measurements were made on the goats including body weight. The goats were machine-milked twice daily (07:30 h and 17:30 h), and during each milking received a concentrate supplementation—the amount of concentrate was calculated to meet the nutritional requirements according to the NRC [25].

### 2.2. Feed Sampling and Analysis

Concentrate and mixed hay were sampled 3 times during the trial: at the beginning, at 3 months, and at 6 months. At each sampling, they were analyzed in duplicate. Dry matter (DM), crude protein, fat, fiber, neutral-detergent fiber, acid detergent fiber, and acid detergent fiber were determined according standard procedures as described by Maggiolino et al. [26]. Non-fiber carbohydrates (NFC) were calculated by subtracting crude protein (CP), ether extract (EE), and neutral detergent fiber (NDF) from the organic matter (OM), and the metabolizable energy (ME) values were calculated using the equation suggested by Robinson et al. [27].

### 2.3. Milk Collection and Analysis

Milk sampling started 3 days after parturition and was carried out by using the same procedure throughout the study 15 days apart until 210 days in milk. A sample of 150 mL was collected at morning and afternoon milking and refrigerated at 4 °C. The 2 samples were pooled according to the relative volume of milk at the afternoon and morning milking to make 1 sample of 150 mL. The sample was immediately transported to the laboratory and analyzed within 2 h for protein and fat. Milk analysis was carried out by infrared spectrophotometry using mid-infrared analysis equipment (Milkoscan FT 6000 milk analyzer, Foss-Electric, Hillerød, Denmark) and in accordance with the guidelines of the International Dairy Federation [28]. Daily milk yield (DMY) was calculated by adding together the morning and afternoon milk yields. Milk yield was standardized at 35 g fat/kg milk according to the following equation [29]:

FCM (3.5%) = DMY * [0.634 + (0.1046 * FY)(1)

in which FCM = fat-corrected milk yield at 3.5% of fat, DMY = milk yield (g/day), and FY = fat content (percentage).

### 2.4. Data and Statistical Analyses

Data were recorded every 15 days on each animal between 15 and 210 days in milk (DIM). A total of 6510 records were obtained for milk yield (g) and fat and protein daily production (g/day). 

All data were tested for normal distribution inside each test day and each breed considered by Shapiro–Wilk test [30].

Two different analyses were conducted.

(1) Wood’s model was applied to average values for each breed to obtain breed curve parameters. It was applied to 3 different test day milk recordings. Firstly, (A) it was applied to all available milk recordings (14 test day milk recordings every 15 days until 210 milking days; (B) to only 1 test for month during 4 months day milk recordings (first, second, third, and fourth month record), and (C) on other 1 test for month during 4 month recordings (first, second, fourth, and sixth month record). Average daily milk yield, FCM, and composition (protein and fat yield) were used to test Wood’s model [14]:

Yt = at^b^e^−ct^ (2)

where Yt is the observed milk yield (g) at day t, e is the Neper number, a is the initial milk yield, b is the rate of increase until the peak is reached, and c is the rate of decline after peak production.

(2) Wood’s model was applied also on the same parameters at the same test day milk recordings (A, B, and C) of each animal in the study. The total milk yield in each goat was calculated for A, B, and C.

Moreover, the total milk yield, fat yield, and protein yield were calculated following ICAR [23] guidelines:

[(Pn + P(n − 1))/2] × d(3)

In which Pn is the production at control n, P(n − 1) is the production at the previous control, and d is the number of days between the two controls. This calculation was done for each interval between 2 monthly controls and then added together to obtain the total milk production.

After this, all parameters were subjected to analysis of variance (ANOVA) using General Linear Model (GLM) of SAS [31], according to the following model:

y_i_ = μ + C_i_ + ε_ij_ (4)

where y_i_ represents the milk, fat, lactose, or protein yield; C is the effect of the ith calculation model (i = 1, 2, 3, 4); and ε_ij_ is the error term. After that, a post hoc comparison by Dunnett’s test [31] comparing to A yield all the others was carried out.

## 3. Results

Table 2 reported *R*^2^ values. Wood’s models showed a good fit in all breeds, showing high *R*^2^ values for all parameters considered in all test-days considered.

Table 3 reported differences between the A Wood’s model application and the other curve calculation methods considered. Considering total milk yield estimation, only B protocol showed higher values than A protocol during lactation in Garganica, Girgentana, Jonica, Maltese, and Saanen (*p* < 0.01). Moreover, in Red Mediterranean, the B protocol estimation showed lower values than A protocol (*p* < 0.01). No statistical differences, in all investigated breeds, were reported for total milk yield estimation between A and C protocols (*p* > 0.05). Regarding FCM, ICAR protocol showed higher values than A protocol only in Maltese (*p* < 0.01) and Saanen (*p* < 0.05) breeds. The B protocol showed higher values in all breeds (*p* < 0.01), except for Jonica, which showed lower values (*p* < 0.01). The C protocol showed higher values only in Maltese and Saanen goats (*p* < 0.01). Fat yield calculated by ICAR protocol in Jonica goats was lower (*p* < 0.01) compared to A protocol. Differently, calculated by B protocol, fat yield resulted in higher A protocol calculation in Garganica, Girgentana, Maltese, and Red Mediterranean goats (*p* < 0.01). No differences reported comparing C protocol to A protocol (*p* > 0.05). Considering protein yield, ICAR and C protocol estimation showed lower values in Jonica goats (*p* < 0.01) compared to A protocol; instead, the B protocol estimation showed higher values in all breeds (*p* < 0.01), except Jonica (*p* > 0.05).

## 4. Discussion

In the present study, we use of Wood’s model to fit lactation curve in six goat breeds in Italy, obtaining high *R*^2^ values. The A protocol showed values ranging from 92% to 95% for milk yield and over 76% for other parameters (fat and protein). This indicated good performance of Wood’s equation in milk-productive trait estimation in goats, considering the 14 test day milk recordings every 15 days during the entire lactation. However, applying Wood’s model to only four test-day milk recordings, we found good fitting for milk yield, with *R*^2^ values over 81% for B and over 84% for C. Moreover, for fat and protein yield, we observed a good fitting of both B and C protocols, except for the Red Mediterranean, in which protein and fat showed *R*^2^ values of 62% and 48%, respectively, for B protocol. High *R*^2^ values are consistent with earlier reports of Saanen [32], Alpine [33], Murciano-Granadina [19,34], Anglo-Nubian [35], and Zaraibi goats [1], in which all authors observed a good fitting of Wood’s model in goat lactation curves. Applying Wood’s model to only the four first-monthly test-days (A) compared to its application to all the 14 test day milk recordings (B), we found a higher milk yield estimation in all goat breeds considered, except for Red Mediterranean, in which we observed an under-estimation. Differences in lactation curves, and thus in terms of Wood’s parameters, could be influenced by genetic makeup of goats, as just observed in other studies [20,36], giving different results in different breeds. However, no differences were reported when comparing the ICAR and C protocol estimations with the A protocol application of Wood’s model. Some authors have reported that Wood’s model tended to over-predict milk production during the early and late lactation period, but to under-predict it during the mid-lactation period in dairy cows [37]. In light of this, it could be that the better distribution of test-day milk recordings (first, second, fourth, and sixth month vs. first, second, third, and fourth month) through the entire lactation gives milk yield estimation nearest to the Wood’s model applied to all available test-day milk recordings. The structure of data analyzed, as the distance from parturition to the first control or the frequency and distribution of records, can result in differences in Wood’s parameters and thus in total yield of the curves [1,19,35,36]. Moreover, for fat and protein total yield, the same trend can be observed. ICAR protocol estimation of fat and protein yield was really similar to A protocol, except for the Jonica breed, in which there was an under-estimation. The results were the same for the C protocol, in which we obtained equal fat and protein total yield in all breeds, except for lower values in protein yield of Jonica breed. Differently, applying the Wood model to the first 4-monthly test-day milk recording (B), we obtained results different in all breeds, except for the Jonica breed, with an over-estimation of fat and protein total yield. Many authors have observed how the goodness of fit depends on the underlying structure of the data, such as the lactation duration or the homogeneity of data recordings [10,37]. The application of the Wood model to different test day milk recordings, considered on the same lactation length, but with different distribution, gives different total milk and milk constituent yield estimations. These results showed how applying Wood’s model on only 4 test-days that were well distributed in the lactation period, we were able to reduce milk yield controls in synchronized flocks for kids production. The study of lactation curve parameter and shapes is useful to characterize the performance of local adapted breeds that normally has a smaller peak of production but more persistent lactation and metabolite concentration, which is desirable because it is related to better animal health and reduction of the feeding costs [5].

## 5. Conclusions

Results show that Wood’s mathematical model fit well on lactation curves of all goat breeds considered. Moreover, applying Wood model to different milk test day recording protocols gave good results and fitting. Observing this, we found that National Breeder Association estimation of total milk fat and protein yield during the entire lactation performed by ICAR guidelines was not different from what Wood’s model estimated if applied on 14 test-day milk recordings (every 15 days) and on only 4 milk test-day recordings (C) if well distributed in the lactation. In fact, C protocol showed better results than B protocol. Considering farms that apply the flock synchronization for reproduction, and thus production activity, we see how this could represent an opportunity for breeder associations to take in account the possibility to consider only 4 test-day milk recordings in goat farms, at the first, second, fourth, and sixth months, and the application of Wood’s model in order to obtain the estimation of total milk, fat, and protein yield during lactation for animals inscribed, or to be inscribed, to the genealogical book.

## Figures and Tables

**Table 1:** Chemical and nutritional composition of concentrate and mixed hay utilized throughout the study.

	Concentrate	Mixed Hay
DM ^1^	88.2	89.1
Crude protein, % DM	21.7	15.1
Fat, % DM	3.5	1.9
Non-structural carbohydrates, % DM	42.7	20.7
Fiber, % DM	10.5	29.5
Ash, % DM	9.1	9.5
Neutral detergent fiber, % DM	23.0	52.6
Acid detergent fiber, % DM	9.0	36.6
Acid detergent lignin, % DM	3.3	3.9
Energy, Kcal/DM	1770	1106

^1^ DM, dry matter.

**Table 2:** Estimated Wood’s model *R*^2^ values for milk yield (g/day) and FCM (fat-corrected milk) to 3.5% of fat, protein, and fat (g/day) in six breeds of goat.

	Garganica (n = 78)	Girgentana (n = 81)	Jonica (n = 76)	Maltese (n = 77)	Saanen (n = 77)	Red Mediterranean (n = 76)
	A ^1^					
Milk yield (g/day)	0.9590	0.9335	0.9282	0.9313	0.9605	0.9281
FCM (3.5%)	0.8282	0.9099	0.8907	0.9295	0.8624	0.8473
Protein (g/day)	0.7616	0.8134	0.8802	0.8924	0.8120	0.8219
Fat (g/day)	0.7636	0.8001	0.8866	0.8378	0.8163	0.8729
	B ^2^					
Milk yield (g/day)	0.9655	0.9979	0.9557	0.9557	0.9904	0.8161
FCM (3.5%)	0.9638	0.9942	0.8560	0.8643	0.9773	0.5785
Protein (g/day)	0.9443	0.9528	0.7491	0.7302	0.9390	0.4812
Fat (g/day)	0.9339	0.9833	0.7425	0.6673	0.9821	0.6159
	C ^3^					
Milk yield (g/day)	0.9755	0.9189	0.9036	0.9036	0.9467	0.8489
FCM (3.5%)	0.9862	0.9412	0.7556	0.7405	0.9974	0.8078
Protein (g/day)	0.9959	0.9682	0.7733	0.9362	0.8716	0.8854
Fat (g/day)	0.9676	0.8422	0.6464	0.6987	0.9873	0.6476

^1^ A: Wood’s model applied to 14 test day milk recordings (15 to 210 days in milk (DIM) every 15 days). ^2^ B: Wood’s model applied to four test-day milk recordings (first, second, third, and fourth months). ^3^ C: Wood’s model applied to four test-day milk recordings (first, second, fourth, and sixth months).

**Table 3:** Differences between the total yield (milk, FCM, fat, and proteins) estimated by Wood’s model application on 14 test-day milk recordings and other calculation methods applied. Results are expressed as least square means and standard error of the mean (SEM).

	A ^1^	ICAR ^2^		B ^2^		C ^3^		SEM
	Milk							
Garganica (n = 78)	199.21	207.34	n.s.	217.69	**	200.46	n.s.	20.49
Girgentana (n = 81)	190.54	198.21	n.s.	231.11	**	197.48	n.s.	19.98
Jonica (n = 76)	256.91	269.57	n.s.	284.68	**	263.57	n.s.	19.62
Maltese (n = 77)	262.67	276.36	n.s.	284.57	**	268.39	n.s.	20.37
Saanen (n = 77)	572.73	598.47	n.s.	701.48	**	610.29	n.s.	19.28
Red Mediterranean (n = 76)	208.12	220.88	n.s.	134.82	**	215.08	n.s.	20.55
	FCM							
Garganica (n = 78)	204.89	211.59	n.s.	222.24	**	207.64	n.s.	19.54
Girgentana (n = 81)	207.52	214.38	n.s.	251.28	**	216.17	n.s.	20.32
Jonica (n = 76)	268.98	279.52	n.s.	253.68	**	272.77	n.s.	18.51
Maltese (n = 77)	260.31	285.68	**	349.53	**	289.58	**	17.36
Saanen (n = 77)	558.95	577.57	*	630.27	**	615.52	**	22.31
Red Mediterranean (n = 76)	224.54	229.53	n.s.	280.95	**	235.99	n.s.	19.42
	Fat							
Garganica (n = 78)	7.40	7.68	n.s.	8.39	**	7.76	n.s.	0.65
Girgentana (n = 81)	7.98	8.47	n.s.	9.87	**	8.43	n.s.	0.69
Jonica (n = 76)	13.02	10.67	**	14.85	n.s.	11.93	n.s.	0.88
Maltese (n = 77)	10.87	10.56	n.s.	13.54	**	11.22	n.s.	0.54
Saanen (n = 77)	18.86	18.98	n.s.	17.95	n.s.	19.48	n.s.	0.94
Red Mediterranean (n = 76)	8.12	8.58	n.s.	10.24	**	9.15	n.s.	0.48
	Protein							
Garganica (n = 78)	7.08	7.28	n.s.	8.16	**	7.13	n.s.	0.61
Girgentana (n = 81)	5.94	6.15	n.s.	8.14	**	6.08	n.s.	0.57
Jonica (n = 76)	10.45	9.02	**	10.02	n.s.	9.67	*	0.63
Maltese (n = 77)	8.31	8.56	n.s.	11.78	**	8.67	n.s.	0.67
Saanen (n = 77)	19.13	19.67	n.s.	22.14	**	20.73	n.s.	0.95
Red Mediterranean (n = 76)	7.04	7.59	n.s.	10.18	**	7.45	n.s.	0.68

^1^ A: Wood’s model applied to 14 test-day milk recordings (15 to 210 DIM, every 15 days) taken as reference. ^2^ ICAR: Calculated according to the International Committee of Animal Recordings guidelines. ^3^ B: Wood’s model applied to four test-day milk recordings (first, second, third, and fourth months). ^4^ C: Wood’s model applied to four test-day milk recordings (first, second, fourth, and sixth months). * = *p* < 0.05; ** = *p* < 0.01; n.s. = not significant.

## Data Availability

Not applicable.
